# Preliminary study of pregnancy rates and litter sizes following artificial insemination of boar spermatozoa prepared by colloid centrifugation and hypothermic storage

**DOI:** 10.1038/s41598-025-24586-4

**Published:** 2025-11-19

**Authors:** Reina Jochems, Camilla O. Kristiansen, Elisabeth Kommisrud, Jane M. Morrell

**Affiliations:** 1 Norsvin, Hamar, Norway; 2 Centre for Embryology and Healthy Development, Department of Biotechnology, CRESCO, University of Inland Norway, Hamar, Norway; 3 Department of Clinical Sciences, Swedish University of Agricultural Sciences, Uppsala, Sweden

**Keywords:** Biotechnology, Physiology, Zoology

## Abstract

Stringent efforts are being made to restrict the development of antimicrobial resistance by good antibiotic stewardship in medical, veterinary and environmental health. In the pig industry, considerable volumes of antibiotic-containing extender are utilised in artificial insemination. Separating spermatozoa from bacteria by colloid centrifugation followed by cold storage could provide an alternative to antibiotics in semen extenders, provided that sperm fertility is not affected by this combination of techniques. The objective of this proof-of-concept study was to compare reproductive outcomes in sows inseminated with control sperm samples or with samples prepared by Single Layer Centrifugation (SLC) through a low density colloid and cooled to 4 °C. A further aim was to compare the SLC sperm preparations with and without antibiotics. Sows were divided into three groups for insemination as follows: control semen prepared and stored conventionally (*n* = 16), SLC samples prepared with antibiotics and cold stored (*n* = 18), SLC samples prepared without antibiotics and cold stored (*n* = 18). The yield of spermatozoa was 84–93%, respectively. Farrowing rates were 88% for controls and 89% for SLC samples. Mean litter size was 16.6, 17.5 and 17 for controls, SLC with antibiotics and SLC without antibiotics, respectively. Stillborn piglets per litter were also similar between groups (0.7, 1.4 and 1.4 respectively). In conclusion, the combination of techniques did not adversely affect sperm fertility or reproductive outcomes of inseminated sows, regardless of whether antibiotics were included in the resuspended sperm pellets after SLC.

## Introduction

Antimicrobial resistance (AMR) is an increasing problem globally, creating a situation in which it becomes impossible to treat bacterial infections^1^. Although bacteria are adept at developing AMR and have done so over many millennia^2^, it is clear that overuse and misuse of antibiotics contribute to the problem, enabling the transmission of resistance between bacteria in humans, animals and the environment^3^. Antimicrobial resistance is thus truly a One Health issue^4^. Some examples of misuse in the livestock production sector are that antibiotics were previously used as growth promotors in animal production or to treat viral infections, but a ban on the former and better knowledge of the latter has reduced such superfluous antibiotic use^5^. Thus, according to the European Food Safety Authority, antibiotic usage in the livestock sector has declined considerably^6^, following specific recommendations for prudent antibiotic usage i.e., that antibiotics should be administered only for therapeutic purposes and after establishing sensitivity to the antibiotic.

One non-therapeutic application of antibiotics that is currently continuing is in semen extenders used in the preparation of semen doses for artificial insemination (AI)^7,8^. Artificial insemination is globally the most widely used assisted reproduction technology in animal breeding; it is particularly prevalent in pig production, where most sows are bred by this technique rather than by natural mating^9^. A major advantage of this reproductive technology over natural mating is that there is a lower risk of transmitting disease if the animals do not come in contact with each other and there is no movement of animals between farms for breeding^10^. A further advantage lies in the wider choice of genetics through AI. The benefits to the pig breeding industry are so great that almost all sows in Europe and North America are bred by this method, amounting to many millions of sows being inseminated annually^11^.

Bacteria are present in the ejaculate of most healthy boars, originating either from the epithelium lining the urethra or from contamination during semen collection despite strict attention to hygiene^12^. Some bacteria have an adverse effect on semen quality^13^, causing sperm motility to deteriorate and sperm aggregation to occur^14^. They may even cause disease in sows after insemination^15^. Therefore, antibiotics are added to semen doses to inhibit the growth of bacteria. Until recently, the addition of antibiotics was mandatory, with national and international regulations specifying the type and concentration of antibiotics to be used e.g. Council Directive 90/429/EEC of the European Union^16^, but now a more liberal wording of some regulations suggests that antibiotics should be added as needed, e.g^17^. Since the personnel on semen collection stations do not know the bacterial load of each ejaculate, or even which bacteria are likely to be present, they will continue to use semen extenders containing antibiotics.

Since most of the liquid fraction of the semen dose passes out of the sow by backflow after cervical insemination^18^, vaginal bacteria and environmental bacteria are exposed to the antibiotics contained in semen extenders. Even low concentrations of antibiotics are sufficient to provoke AMR^19^ and resistance to antibiotics was reported in bacteria found in boar semen^20–22^.

Some research into alternatives to inhibit bacterial growth in semen doses has been conducted, e.g. by reducing the temperature^8,23^, since it was previously thought that most of the bacteria in semen would not survive below 15 °C. Subsequent investigation indicated that this is either not the case or that bacteria may indeed stop growing at temperatures below 15 °C but are able to resume growth when the temperature is raised again^24^, for example, when insemination occurs, or after insemination. Therefore, hypothermic storage by itself may not be a feasible alternative to antibiotics in semen extenders, unless the semen was collected with a low bacterial load and is to be inseminated within 24 h. Furthermore, it was previously assumed that cooling below 15 °C was detrimental to boar sperm survival^25^, which is why semen doses are conventionally transported at 17–23 °C and stored at approximately 17 °C^26^. The infrastructure of the pig breeding industry is organised accordingly, using incubators at the appropriate temperature for on-farm storage of semen doses. Since bacteria can rapidly develop resistance to any substance that is used against them, trying to develop alternatives to inhibit bacterial growth may merely lead to more resistance. In this respect, one of the most interesting alternatives to antibiotics is the separation of spermatozoa from bacteria by colloid centrifugation, since such a technique should not induce the development of resistance. Colloid centrifugation was first reported as a potential solution to antibiotic usage in 2011^27^ but there was little interest from pig breeders due to its high cost and the extra work on the semen station. Colloids based on coated-silica particles are expensive because of the raw material. Subsequent research into the use of a low density colloid, Porcicoll, indicated that spermatozoa could be separated from bacteria without any detrimental effect on sperm quality^28,29^ and fertility was maintained in a field trial^30,31^. These results are promising but a more appealing concept would be the possibility of combining colloid centrifugation and cold storage of boar spermatozoa, to reduce the bacterial load and then to prevent multiplication during storage. However, it was not known whether boar spermatozoa could survive cold storage without the protective effect of seminal plasma^32^, since seminal plasma is removed as the spermatozoa pass through the colloid^33^. Therefore, a preliminary study was conducted in which boar spermatozoa prepared by Single Layer Centrifugation (SLC) through a low density colloid were cooled slowly and then stored in the cold room (4 °C) for 4 days; sperm quality (membrane integrity, chromatin integrity and mitochondrial membrane potential) was not different from controls stored at 17 °C^34^, except that chromatin integrity was better in the cold-stored samples after 4 days. However, the fertility of the samples was not determined in this study.

The objective of the present study, therefore, was to determine whether boar spermatozoa, processed by SLC through a low density colloid, Porcicoll, could be cooled to 4 °C and stored, and still retain their fertility when used in AI. A further aim was to investigate whether the omission of antibiotics from the processed samples had a detrimental effect on the reproductive outcomes of the inseminated sows.

## Results

### Yield

The average yield of spermatozoa recovered was 93% in extender without antibiotics and 84% in extender with antibiotics (Table 1); the difference is not significant (*P* = 0.5).

**Table 1:** Yield of Boar spermatozoa (x10^6^) after single layer centrifugation through a low density colloid.

Batch	Sperm concentration in pooled semen	No. spz added to tube	Recovery rate (no antibiotics)	Recovery rate (with antibiotics)
1	142	34,000	31,950 (92%)	30,030 (88.5%)
2	161	38,640	37,000 (95.8%)	31,000 (80%)
3	201	48,240	44,000 (91.2%)	40,300 (83.5%)
Mean ± SD (%)			37,650 ± 6051 (93%)	33,777 ± 5670 (84%)

Note: All concentrations are x10^6^/mL. Approximately 80 mL of each semen pool was layered on top of 60 mL colloid in each of three 200 mL tubes for centrifugation at 300 g for 20 min. The sperm pellets from the 3 tubes were combined and extended in AndroStar Premium either with or without antibiotics. The procedure was performed on three occasions. Spz = spermatozoa. Recovery rate = (no. spz recovered/no. spz in original load) x 100.

### Reproductive data

In the first batch there were 3 returns to oestrus: two in the group SLC without antibiotics, and one in the control group. Of these, two sows were only weakly in heat on the first day of insemination and were not in heat on the second day (one in the SLC group without antibiotics and one in the control). In the second batch, all sows became pregnant although one sow did not farrow (SLC with antibiotics group). All sows in the third batch became pregnant, although two sows did not farrow (one from the SLC with antibiotics groups and one from the control group). Thus, the overall pregnancy rate was 89% for the SLC groups and 88% for the controls (Table 2). However, assuming that the sow that was only weakly in heat at the time of insemination did not become pregnant because of the poor timing of insemination relative to ovulation rather than due to sperm treatment, the pregnancy rate for the group of SLC without antibiotics would be 94%. All eleven sows that were inseminated with one of the SLC groups in the first round and then were inseminated again in the second round became pregnant again (Table 3), indicating that there was no apparent detrimental effect on subsequent fertility. However, one of these sows was subsequently found to be empty at farrowing.

**Table 2:** Reproductive performance for each group of sows (SLC + cold storage without antibiotics, SLC + cold storage with antibiotics, and control with antibiotics).

Type of inseminate	Pregnancy rate (%)	Farrowing rate (%)	Total live born	Liveborn piglets/litter	Stillborn piglets/litter
Without antibiotics	89 (16/18)	89 (16/18) §	272	17 ± 3.2 (272/16)	1.4 ± 1.5
With antibiotics	100 (18/18)	89 (16/18)	280	17.5 ± 3.3 (280/16)	1.4 ± 1.9
Control	88 (14/16)	88 (14/16)	213	16.4 ± 3.2 (213/13) §§	0.8 ± 0.4

Notes: SLC = Single Layer Centrifugation; § One sow was only weakly in heat on the first insemination day and was not in heat for the second insemination. §§One sow was euthanased because of dystocia, so litter size is only calculated for 13 sows. The numbers of liveborn piglets per litter and stillborn piglets per litter were not different between the treatments.

**Table 3:** Reproductive outcomes in sows inseminated with colloid centrifuged, cold-stored semen either with or without antibiotics in more than one round of inseminations (*n* = 9).

Sow	Parity	Insemination dose in round 1	Outcome	Parity	Insemination dose in round 2	Outcome
1	3	Without Ab	Pregnant	4	With Ab	Pregnant
2	3	Control	Pregnant	4	Without	Pregnant
3	2	With Ab	Pregnant	3	Without Ab	Pregnant
4	2	With Ab	Pregnant	3	Without Ab	Pregnant
5	2	Without Ab	Pregnant	3	With Ab	Pregnant
6	2	Control	Pregnant	3	With Ab	Empty
7	2	With Ab	Pregnant	3	Control	Pregnant
8	2	Without Ab	Pregnant	3	Control	Pregnant
9	1	With Ab	Pregnant	2	Control	Pregnant
10	1	With Ab	Pregnant	2	Without Ab	Pregnant
11	1	Without Ab	Pregnant	2	With Ab	Pregnant

Note: Ab = antibiotics; empty = sow was diagnosed as pregnant after artificial insemination but was not pregnant at farrowing.

## Discussion

The objective of this study was to determine whether boar spermatozoa, processed by SLC through a low density colloid, could be cooled to 4 °C and still retain their fertility when used in AI. A further aim was to investigate whether the omission of antibiotics from the processed samples affected the reproductive outcomes of the inseminated sows. The results showed that pregnancy rate and farrowing rate were not different between the controls and the SLC plus cold storage groups, regardless of whether the latter contained antibiotics or not. The numbers of liveborn and stillborn piglets, and the sex ratio, were not different between the treatment groups.

Although previous publications reported pregnancy and farrowing rate from sows inseminated with boar sperm samples prepared by SLC without antibiotics that were not different from controls, the samples were maintained at 17 °C [29, 30]. There is also a publication reporting pregnancy rates from cold-stored boar semen without antibiotics^35^, although the samples were not prepared by SLC. Thus, the present proof-of-concept study is the first time that the combination of SLC and cold storage has been attempted, with or without antibiotics, using conventional sperm numbers in the insemination doses. The results in terms of pregnancy rates, farrowing rates and number of piglets per litter, which were not different from controls^36^, are very encouraging, since it appears that the presence of seminal plasma is not required to protect boar spermatozoa from cold shock when extended in AndroStar Premium. This result is in contrast to a previous study where seminal plasma was thought to protect spermatozoa during cooling prior to freezing^32^. The observations that antibiotics could be omitted from the extender without any detrimental effect on the pregnancy rate or on litter size, or on the subsequent fertility of the sow, are extremely pertinent to the quest for alternatives to antibiotics in semen extenders.

Two sows in the first batch were thought to be only weakly in heat on the first insemination day. They were not in heat on the second insemination day and therefore did not receive a second insemination. One of these sows did become pregnant (control group) but the other (SLC without antibiotics) did not. It is not known whether the latter sow did not conceive because of insemination at the wrong time relative to ovulation. Two sows that were diagnosed as pregnant at the first pregnancy check were subsequently found to be not pregnant at farrowing (“empty”). One of these sows was in the control group for the first round of inseminations and in the SLC cold storage with antibiotics group in the second round. Muirhead & Alexander^36^ reported that 5 to 15% of inseminated or mated sows are empty, and that 4% of sows diagnosed as pregnant do not farrow. In the present study, 5 out of 54 inseminated sows were empty (10%); 2 out of 48 sows diagnosed as pregnant did not farrow, which corresponds to the 4% pregnancy loss reported by Muirhead & Alexander^37^. Published reports for this hybrid suggest litter sizes depend on parity, with Hales suggesting an average litter size of 14.7 for parity 1 sows, 16.9 for parity 2 sows, and 18.2 for sows in parity 3–4^38^. Kobek Kjelldager et al. observed litter sizes of 15.3, 16.4, 16.5 and 19.5 for parities 1, 2, 3, and 4, respectively^39^. The previous parities of the sows in this study (14.8 ± 2; 15.1 ± 3.5, 16.3 ± 2.5, 20 ± 3.6 for parities, 1, 2, 3, and 4 respectively) correspond well with these values, as do the total born for the three groups (17.5, 18.9 and 18.4 for controls, SLC with and without antibiotics, respectively).

The control samples were stored at 14 °C, which is the routine procedure on this farm. On other farms, the conventional storage temperature is 17 °C^11^ although some reports suggested that extended semen doses can be stored at temperatures as low as 10 °C without loss of function^40^. In another study, acceptable fertility rates could be achieved under storage conditions as low as 12 °C for 48 h in Androhep extender^25^. In contrast, storage temperatures below 20 °C were reported to affect sperm motility and acrosome integrity in Norwegian Landrace boar semen^41^, although sperm survival during storage at 10 °C varied considerably between individuals. In any case, the reproductive outcomes from inseminations with the SLC cold-stored samples in the present study were not worse than the controls and corresponded well to the average total born per litter for Durocs in a Danish study, which was reported to be 18.2^42^.

A previous study in which sows were inseminated with SLC-prepared boar sperm doses without antibiotics in more than one pregnancy indicated that there was no detrimental effect on subsequent fertility in the sows^43^. Our present results, albeit with a small number of sows, tend to corroborate these findings. A study in a larger number of sows on different farms should be undertaken to substantiate these results. An AI trial in Brazil used cold-stored semen after 120 h storage without antibiotics, but the sperm samples were not prepared by SLC before cooling^35^.

The present cooling protocol involved holding the samples at ambient temperature during the processing, extension and packaging of the semen doses (approximately 3 h) followed by cooling from ambient temperature (20 °C) to 4 °C in 6 h. The latter represents a cooling rate of 0.04 °C/min, which coincidentally corresponds to the revised cooling rate reported by Luther et al.^44^, albeit after a longer holding time at 17 °C before cooling. Previously their group had used a slower cooling rate and a longer holding time at 17 °C^8^.

The advantages of cold storage of boar semen over the conventional storage temperature have been reported previously^34^, including temperature control during transport and avoidance of a rise in sperm DNA fragmentation during storage^45^. Maintaining the optimal temperature for boar semen during transport in hot countries is notoriously difficult, and there is typically a loss in productivity during the hottest months of the year^46^. Sub-optimal temperatures during the transport of semen doses may contribute to this problem. Maintaining the temperature of the semen during transport may also be a problem in cold countries during the winter. Prolonged temperatures above 23 °C are associated with energy expenditure and the formation of metabolic byproducts that are detrimental to sperm survival^47^. On the other hand, boar spermatozoa stored at 5 °C may use considerable amounts of ATP to become reactivated when warmed to body temperature^48^, as occurs when the semen dose is inseminated. However, the latter authors commented that there may still be sufficient spermatozoa with enough energy to move through the sow´s reproductive tract and undergo capacitation and the acrosome reaction. This would certainly appear to be the case in the current study, where pregnancy rates, farrowing rates and litter sizes were similar in controls and cold-stored sperm samples.

The results presented here in this proof-of-concept study suggest that boar semen doses prepared by colloid centrifugation and then cooled to 4 °C generate the same pregnancy rates and litter sizes as conventionally stored semen doses. There was no difference between the SLC-samples containing antibiotics and those without antibiotics, suggesting that for these semen samples there is no need to add antibiotics to the semen extender. Although bacterial counts were not done in the present study, a previous experiment with Hampshire boar semen showed that there was no difference in bacterial counts between samples stored conventionally with antibiotics at 16–18 °C and those processed by SLC, cooled slowly and stored at 4 °C for 4 days^34^. The difference to the present study was that Ngo et al.^34^ used semen that was transported from the boar station to the laboratory for approximately 4 h before SLC, whereas in the present study the semen was delivered to the laboratory within 1 h of semen collection. Conducting bacteriology on semen doses stored on the farm was not possible in the present study but will be included in future AI trials. However, we have previously conducted studies on bacteria in semen from several species processed by SLC, showing a considerable reduction in bacterial load (e.g., 90–100%^14,27,28^. Based on the results of this preliminary study, further AI trials can now be planned. Future studies will include different storage times, other breeds, larger numbers of sows, and different husbandry conditions in different countries, as well as microbiology of the samples. The current study used pooled semen samples; the small number of sows available did not permit semen from individual boars to be used, since this would have added another source of variation. However, individual variation between boars should also be explored.

The SLC with antibiotics group was included in the experimental design as an additional control. If there had been a difference in performance between the controls and SLC group without antibiotics, we would not have known if this was due to sperm treatment or to the absence of antibiotics. Therefore, the SLC with antibiotics group was added.

Since the insemination dose in the sow is usually 80–90 mL and each sow is inseminated twice, considerable amounts of antibiotic-containing semen extender are currently used in pig AI. Most of this liquid is subsequently expelled into the environment when cervical deposition of semen is used, exposing environmental bacteria to the antibiotics. Refraining from adding antibiotics to semen extenders could thus help to slow the development of AMR, especially in the major pig-producing countries. Obviously, such a paradigm-shift in the way in which boar semen is prepared and stored for insemination can be challenging but, at the same time, urgent action is needed to reduce AMR if the considerable benefits to be derived from AI are to continue. Refrigerated transport is already available for other products, it would not be too difficult to transport semen doses in the same manner^49^. Thus, it is the routines at the semen collection center and current on-farm storage and handling that would need to be changed.

## Conclusion

The results of the present study indicate that cold storage of boar semen after SLC has the potential to provide a useful alternative to antibiotics in semen extenders, with important implications for combatting development of antimicrobial resistance and for One Health. The combination of techniques does not adversely affect sperm fertility or reproductive outcomes of inseminated sows. There were no differences in reproductive outcomes after this combination of treatments regardless of whether or not antibiotics were included in the extender to resuspend the sperm pellets. A larger field trial is warranted, incorporating pigs kept on different farms under different conditions in various countries.

## Methods

**Fig. 1:**
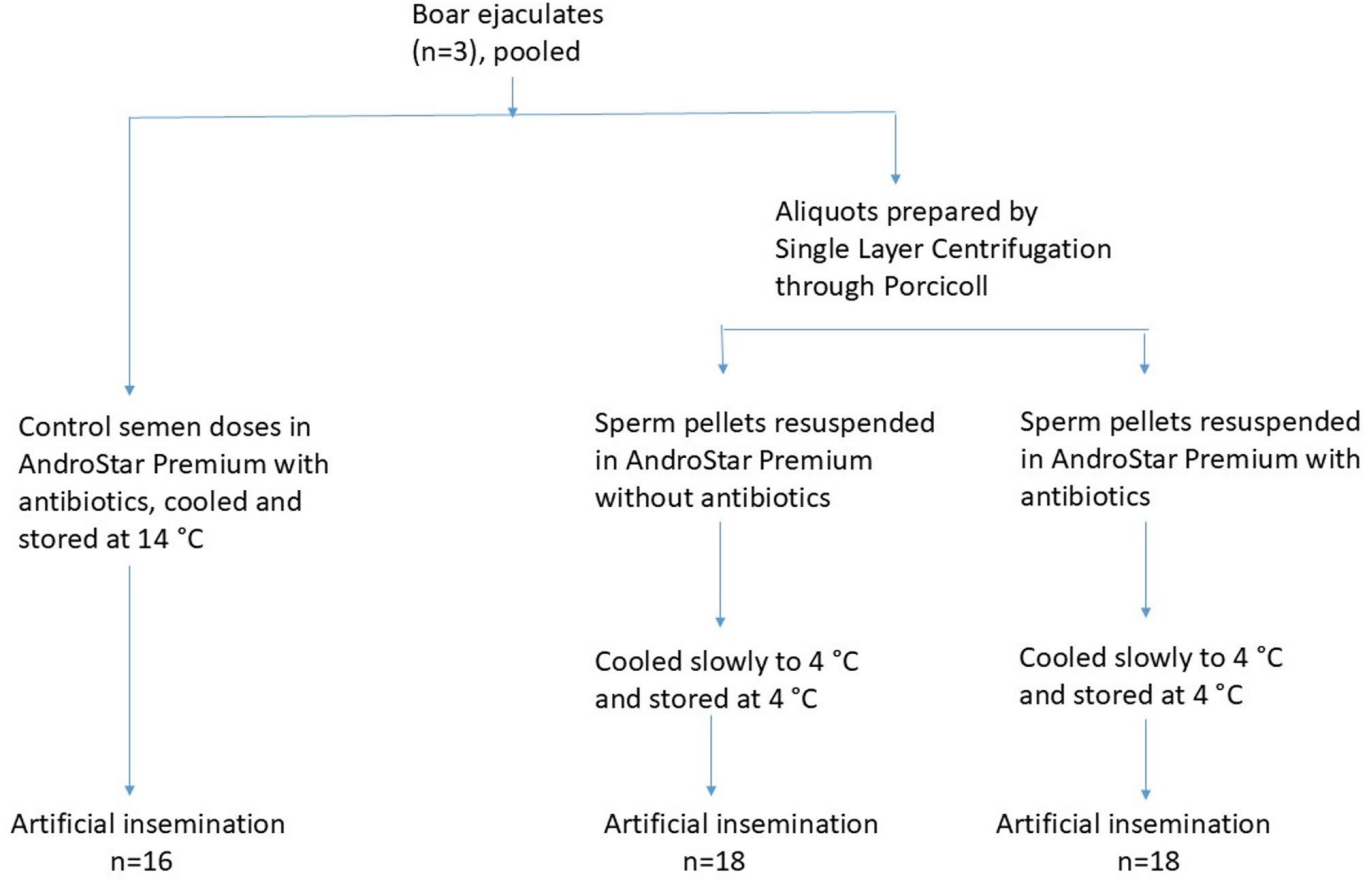
Experimental design. Note: the semen collections and preparation were repeated to give a total of three rounds of inseminations.

The experimental design is depicted in Fig. 1.

### Boar housing and management

The boars were housed at a commercial boar semen collection station (Norsvin, Hamar, Norway) according to national and international regulations on the husbandry and care of livestock, in individual pens of floor area 6.2 m^2^. The temperature was maintained at 17–18 °C, either by supplying heat in the winter or cooling by high pressure spray mist in the summer, which also has the function of reducing dust. They were fed a commercial diet according to age and body condition, amounting to 2.8–4.0 kg feed per day. The diet contained 14% crude protein, with net energy 9.68 MJ per kg; the standard ileal digestible lysine content was 7.7 g per kg. In addition, each boar was provided with sugar beet pulp daily and rooting material in the form of hay or straw; they had access to clean water *ad libitum*. There were 270 boars on the station at the time of the study, with an age range of 9 months to 4 years.

### Semen collection and preparation of semen doses

All handling of the boars and semen collection was done by Norsvin personnel. Semen collection by the gloved-hand method is regarded as a normal husbandry procedure for pigs and does not require ethical approval. Semen was collected twice weekly on a routine basis as follows: when the boar mounted the dummy sow, the abdominal skin and preputial opening were cleaned with paper and the preputial diverticulum was emptied of fluid. Semen was collected using the gloved hand technique^9^. The first (clear) fraction was discarded; when production of the sperm-rich fraction began, the semen was collected into a paper collecting cup covered with a filter to retain the gel. Once the collection was finished, the filter (and gel) was discarded, the semen was poured into a warm (35 °C) bottle and sent by tube mail to the laboratory. Sperm motility and morphology were evaluated at 37 °C using an IVOS II (IMV Technologies, L’Aigle, France) and Leja slides (Leja, Nieuw-Vennep, The Netherlands). Ejaculates with < 75% motile and/or > 20% morphologically abnormal sperm were discarded. The morphological defects included head defects, tails defects and proximal cytoplasmic droplets. The study took place on three occasions between May and December 2024; on each occasion, semen was collected from three Duroc boars. The average age of these boars was 26.3 ± 9.6 months. The three ejaculates, each with > 75% total motility and < 20% morphologically abnormal spermatozoa were pooled; sufficient semen was removed from the pool to make up conventional semen doses as controls, using AndroStar Premium with antibiotics at 25 °C. Each dose contained 1.8 × 10^9^ spermatozoa in 90 mL, packaged in plastic semen bags. These semen doses were subsequently kept at approximately 16 °C, while the remainder of the pool was being processed. The semen doses were transported to the University of Inland Norway, Hamar, Norway (INN) at ambient temperature for approximately 20 min and were then stored at 17 °C while the rest of the semen was being prepared.

The semen pool was diluted 1:1 in AndroStar Premium without antibiotics at 25 °C and was transported to the laboratory at the University of Inland Norway, Hamar, Norway, in an insulated box at ambient temperature. In the laboratory at INN, the semen was used for Single Layer Centrifugation through low density (20%) Porcicoll at 18 °C using 60 mL colloid and 80 mL semen per 225 mL tube^50^. Pairs of tubes were prepared. The tubes were centrifuged at 300 g for 20 min at ambient temperature before removing the supernatant (seminal plasma and colloid) with a vacuum pump. The sperm pellet was resuspended in AndroStar Premium extender with or without antibiotics at ambient temperature (approximately 20 °C) so that insemination doses in both types of extender could be prepared. The recovery rate (%) was calculated by dividing the number of spermatozoa obtained in the sperm pellet by the number of spermatozoa added to the tube, multiplied by 100. The sperm concentration was determined by CASA (Sperm Class Analyzer^®^, version 6.1, Microptic SL, Barcelona, Spain) and adjusted to provide insemination doses of 1.8 × 10^9^ spermatozoa in 90 mL extender, which were packaged in colour-coded semen bottles (Minitüb International, Tiefenbach, Germany). The bottles were placed in an insulated box in the cold room at 4 °C, with a thermometer in the box. The temperature was checked every hour until the contents of the box had reached 4 °C, at which time the bottles were transferred to an insulated carrying box containing cold packs for transport to the farm. These cold packs had been kept at −20 °C overnight and were wrapped in paper towels to prevent direct contact with the bottles containing the semen doses. The control semen doses were transported to the sow farm in a conventional boar semen transport box at ambient temperature, separately from the cold-stored semen.

At the farm, the control semen doses were stored at 14 °C (their usual storage temperature for boar semen), whilst the cold-stored semen remained in the transport box with the cold packs. Further cold packs were added when the first insemination doses were removed, to maintain the temperature at approximately 4 °C. The temperature was checked with a thermometer before the semen doses were removed for AI. Artificial insemination was carried out approximately 24 and 48 h after semen collection and preparation, by the barn staff at the farm.

### Sows and artificial insemination

Sows (TN70 hybrids) were purchased as first time breeders and remained on the farm for several parities. They were wormed and vaccinated regularly, according to national and international recommendations. Sows were group-housed in controlled environment at 16 °C on straw bedding under a lighting regimen of 17 h light, 7 h dark. Fresh straw and shavings were added as required. They were transferred to individual pens for farrowing. After farrowing, they received a commercial farrowing diet ad libitum and creep feed was available for the piglets. After weaning, the sows were fed 3 kg of a commercial maintenance feed each day (1.5 kg twice daily), although thin sows were given more feed to restore body condition. A breakdown of the different diets is shown in Table 4. Fresh water was always available.

**Table 4:** Composition of the commercial diets available at different stages.

Ingredient	Creep feed	Sow feed	Sows after weaning
Crude protein	17.8%	14.7%	12.4%
Fat	4.6%	4.6%	3.7%
Crude Fibre	3.5%	5.1%	9.6%
Ash	5.4%	4.9%	4.4%
Calcium	8.3 g	7.3 g	6.3 g
Phosphorous	5.0 g	4.6 g	3.7 g
Magnesium	1.3 g	1.4 g	1.4 g
Sodium	2.3 g	2.0 g	2.0 g
Lysine	13.7 g	8.8 g	5.8 g
Methionine	4.4 g	2.5 g	1.9 g

Sows were fully vaccinated against parvo, leptospirosis and erysipelas, and were dewormed before weaning. The vaccinations were administered to replacement gilts before entering the sow barn. All females were vaccinated against *Escherichia coli* two weeks prior to farrowing.

The average weaning-to-estrus interval on this unit was 5 days, The females were checked for signs of estrus daily on days 2 and 3 after weaning, and then twice daily for four days. The response to the presence of a boar was checked as well as the back-pressure test. Artificial insemination was carried out on days 5 and 6 after weaning, if the sows were in standing heat. (It is standard practice on the farm that any sows still in estrus on the day after the second insemination would be inseminated a third time, but this did not occur in any of the sows in this study).

The overall statistics for the pig unit are as follows: mean farrowing rate, 90.5%, average litter size, 16.1 and average number of stillborn piglets per litter, 1.

For this study, sows were divided into three similar groups, matching parity where possible. Sow selection was based on number of farrowings and number of piglets produced, as well as body condition score. They were tested for standing heat each day from day 2 after weaning Each sow was inseminated twice, provided they were still in standing heat on the second occasion^11^.

In total, three batches of sows were inseminated. The first batch consisted of 17 sows; the second batch also contained 17 sows, eleven of which had been inseminated in the first batch, had farrowed and their litters were now weaned. Six gilts were included to replace the older sows. The third batch consisted of 18 sows, which had not been included in the study in the previous batches. All sows were checked for signs of oestrus 21 days after insemination; those that showed heat were deemed to be not pregnant. Sows that did not show oestrus were checked for pregnancy one month after AI.

### Data recording

Sow identity and type of semen inseminated were recorded on the sow cards in the barn and in the farm database. Any returns to oestrus 21 days after insemination were reported. Pregnancy checks with ultrasound were conducted one month after AI and results recorded. At parturition, sows were kept under observation so that assistance could be provided in case of dystocia. The number of piglets born, live or stillborn, was recorded. Data were made available to the authors from the computer records.

Ethical permission was not required for this study since semen collection and AI are routine husbandry procedures and were carried out with the permission of the owner. All pigs were handled and cared for according to internationally recognized guidelines and regulations for keeping pigs in Norway (The Animal Welfare Act^51^; and Regulations for keeping pigs in Norway^52^.

#### Statistical analysis

Chi-Squared test was used to compare the pregnancy rates and farrowing rates between the three experimental groups. One-way analysis of variance (ANOVA) was used to compare the number of liveborn piglets and stillborn piglets born in the three groups. The level of significance was set at *P* < 0.05.

## Data Availability

All data generated or analysed during this study are included in this published article.
